# Planar ultrasonic transducer based on a metasurface piezoelectric ring array for subwavelength acoustic focusing in water

**DOI:** 10.1038/s41598-022-05547-7

**Published:** 2022-01-27

**Authors:** Shin Hur, Hyunggyu Choi, Gil Ho Yoon, Nam Woon Kim, Duck-Gyu Lee, Yong Tae Kim

**Affiliations:** 1 Department of Nature-Inspired System and Application, Korea Institute of Machinery and Materials, 156 Gajeongbuk-Ro, 34103 Daejeon, Republic of Korea; 2 Acoustics, Ultrasound and Vibration Group, Korea Research Institute of Standards and Science (KRISS), 267 Gajeong-ro, Yuseong-gu, Daejeon, 34113 Republic of Korea; 3 School of Mechanical Engineering, Hanyang University, 222, Wangsimni-ro, Seongdong-gu, Seoul, Republic of Korea; 4 University of Science and Technology, 217, Gajeong-ro, Yuseong-gu, Daejeon, 34113 Republic of Korea

**Keywords:** Engineering, Physics

## Abstract

The development of a new ultrasonic transducer capable of improved focusing performance has become a necessity to overcome the limitations of conventional ultrasonic transducer technology. In this study, we designed and optimized a metasurface piezoelectric ring device, and using multiphysics finite element analysis, we examined the performance of a planar ultrasonic transducer consisting of this device, a matching layer, a backing layer, and housing in producing a needle-like subwavelength focusing beam in water. For practical experiments, a metasurface piezoelectric ring device was fabricated using a laser ablation process. Subsequently, using a pulse-echo test, we found that the − 6 dB bandwidth of a planar ultrasonic transducer with a center frequency of 1.0 MHz was 37.5%. In addition, the results of an ultrasonic-focusing performance test showed that the full width at half-maximum of the axial subwavelength focusing beam was 0.78λ, and the full lateral width at half-maximum of the subwavelength lateral focusing beam was 7.03λ at a distance of 10.89λ. The needle-like focused ultrasonic beam technology implemented with a piezoelectric ring array based new planar ultrasound transducer is expected to be used in high-resolution imaging devices or medical ultrasound focusing devices in the future.

## Introduction

**Figure 1:**
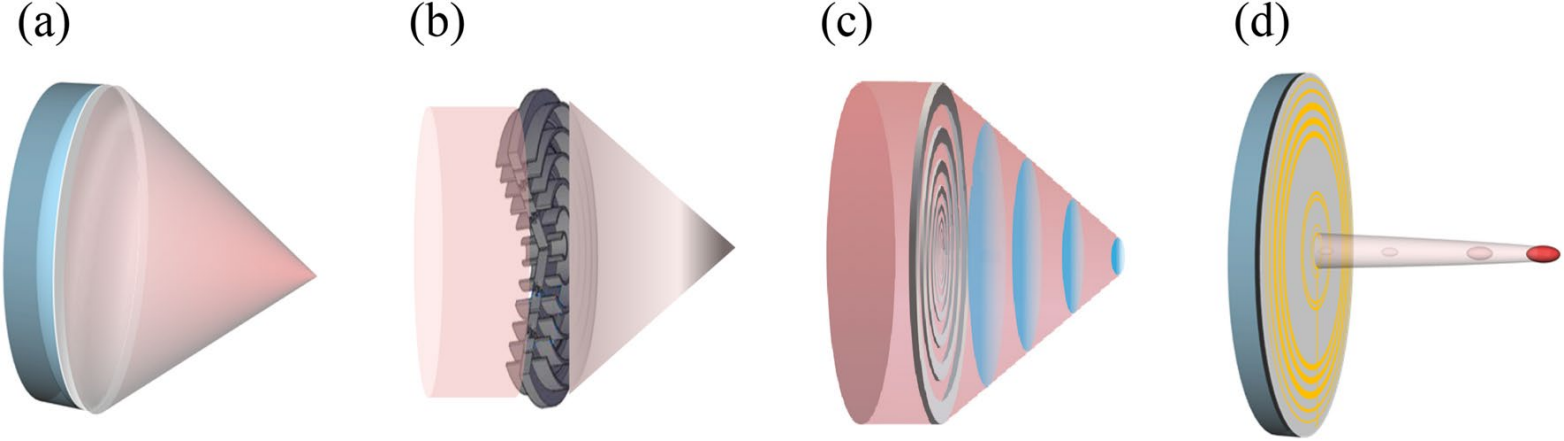
Illustrations of a range of acoustic-focusing transducer technologies (AutoCAD Mechanical Desktop 2006, Version 2006, RUL: https://mechanical-desktop-6.software.informer.com): (**a**) curved piezoelectric ultrasonic transducer. (**b**) acoustic lens based on space-coiling metamaterial. (**c**) acoustic lens based on flat annular ring array. (**d**) planar piezoelectric ultrasonic transducer based on piezoelectric ring array.

Ultrasonic transducers are based on the direct and indirect effects of piezoelectric materials to generate mechanical energy in response to electrical signals and, conversely, electrical signals in response to mechanical vibrations^1–6^. Owing to characteristics such as their wide bandwidth, fast response, and high sensitivity, devices and applications based on ultrasonic transducers, such as ultrasonic imaging^7^, acoustic tweezers, intravascular therapy^8,9^, and surgical ultrasound tools, have been developed. The increasing demand for microimaging and precise manipulation of microparticles in bio tissues has led to the study of higher frequencies, device miniaturization, and new features for ultrasonic transducers^10,11^. With a conventional ultrasonic focusing device, as shown in Fig. 1a, the size, shape, distance and intensity of the focused beam are determined only by the curved transducer structure, i.e., hardware technology, without beamforming algorithms and complex signal processing techniques. The focusing resolution produced by these curved piezoelectric transducers (PTs) is usually low, and the focal size is relatively larger than one wavelength. Although increasing the driving frequency can improve a transducer’s focusing resolution, driving at higher frequencies results in higher energy consumption because the focused beam suffers stronger attenuation. In addition, acoustic aberrations can significantly reduce the focusing resolution. Hence, a variety of approaches have been investigated to address the aforementioned issues.

The result of the initial research into planar subwavelength beam focusing is a technology implemented with linear array acoustic transducers^12^ based on the shift beam principle, a near-field antenna array theory developed for subwavelength focusing of electromagnetic waves. Also, several studies have been conducted focusing on acoustic lens based on space-coiling metamaterial (Fig. 1b), resulting in the development of acoustic lenses with resonant extraordinary acoustic transmission (EAT)^13^; directional metasurfaces consisting of an array of subwavelength Helmholtz resonators with differing internal coil path lengths^14–16^; and acoustic metasurface composed of spatial-coiling subunits that generate two symmetrical parabolic accelerating beams^17^. Flat annular Fresnel piezoelectric transducers, which reduce the volume of piezoelectric material used in fabrication, were developed in a separate approach to prevent the volume of curved PTs from becoming too large. However, because Fresnel PTs are always accompanied by higher order diffraction, they cannot fully concentrate on the acoustic energy generated. Hence, plane wave generating metasurface lenses, consisting of a thin plate structure with a perforated deep subwavelength slit separated from the sound source, were developed to perform far and near field sound focusing through wave manipulation^18–20^. A technology that implements a focusing beam by reflecting the incident wave on a planar metasurface is shown in Fig. 1c. Here, a high gain acoustic antenna forms an acoustic beam using an admittance-modulated metasurface composed of periodic subwavelength grooves with a sinusoidal depth profile, and the periodic groove profile can be modulated as an independent binary metasurface to control the beam angle and beamwidth^21–23^. Recently, using optimized design methods^24,25^, advanced ultrasonic acoustic-focusing devices with simplified architectures have been developed to achieve subwavelength imaging and active acoustic focusing. A planar ultrasonic transducer (PUT) for focusing acoustic waves below the wavelength is shown in Fig. 1d.

Beyond investigation of planar metasurfaces for ultrasonic transducer technology, studies focusing on engineering piezoelectric materials have been conducted, from which several ultrasonic devices have been developed. These include an annular interdigital transducer that concentrates acoustic waves on the surface of lithium niobate to a single point^26^; an ultrathin helical 3D piezoelectric element for generating arbitrarily complicated ultrasonic fields^27^; and active acoustic metasurface consisting of 16 × 16 square lattice elements of subwavelength thickness in which each element is a supercell with a 4 × 4 piezoelectric sheet embedded in a matrix of epoxy resin^28^ capable of eliminating the grating lobes caused by structural diffraction and steering the ultrasonic focus. Additionally, design optimization of piezoelectric ring array for PUT was carried out for manipulating acoustic focus patterns and focusing resolution freely^29^. Since a piezoelectric ring array composed of a flat thin layer has the properties of a metasurface, we defined it as a metasurface piezoelectric ring array (MPRA). A planar PUT integrating such an MPRA realizes a subwavelength ultrasonic focused beam^30^.

When engineering piezoelectric materials, laser ablation methods are found to be more convenient than conventional deep reactive ion etching (DRIE), which takes a long time and requires hard mask deposition for rapid micromachining of complex patterns with precision. Studies in which piezoelectric materials have been processed using laser ablation include one in which PVDF films were patterned with a 193-nm excimer laser to create bioelectronic devices^31^, an investigation into the creation of electrodes on piezoelectric ceramics for sensor applications using ns-pulsed laser ablation^32^, a comparative study on the machinability of lead magnesium niobate–lead titanate using excimer laser ablation and DRIE^33^, and a study on the growth and deposition process of laser-ablation-deposited piezoelectric thin films for piezoelectric microdevices^34^. However, reports on micromachining of complex piezoelectric patterns in an ultrasonic transducer are comparatively few. Moreover, there have been no experimental demonstrations of a fabricated PUT capable of subwavelength acoustic focusing that overcomes diffraction limitations.

In this study, we developed a method for optimizing metasurface piezoelectric ring array(MPRA) capable of forming a needle-like subwavelength ultrasonic-focusing beam and prepared a detailed structural design for a PUT consisting of a matching layer, MPRA layer, backing layer, and housing using multiphysics finite element analysis. An MPRA with complex patterns was fabricated by establishing an optimal laser ablation fabrication process, and the complete PUT was manufactured following a final packaging and assembly process. Subsequently, the device’s center frequency and frequency band were measured using a hydrophone. Finally, the performance of the subwavelength ultrasonic-focusing beam was tested and compared with the result of the simulation.

## Simulation study

As wave propagation depends on the material medium, a medium-based design is required to ensure that the acoustic-pressure-focusing device can be used on the human body. However, verifying structures directly on the human body is problematic. Therefore, we devised an optimization experiment with a water set as the propagation medium, as the impedance of water is similar to that of the human body. The MPRA of the PUT was designed using sensitivity-based topology optimization, a technique to produce features that are difficult to manufacture. To solve this problem, we completed optimization using a rectangular filtering condition with a constant height. For acoustic-pressure-focusing at a frequency of 1.0 MHz, the speed of sound in water is 1496 m/s.

**Figure 2:**
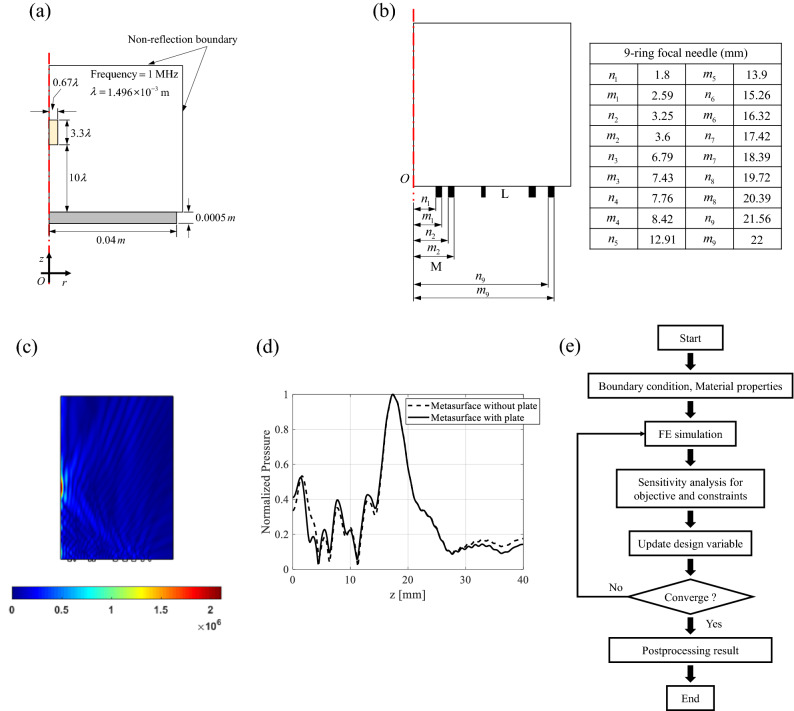
Planar piezoelectric ultrasonic transducer (PUT) applying the design method of the reference^30^: (**a**) a problem definition of PUT to the 2D axisymmetric finite element simulation, (**b**) optimized structure and distance of piezoelectric ring (inner radius of n and outer radius m), (**c**) response of absolute pressure to the piezoelectric ring, (**d**) acoustic focusing response of the patterned piezoelectric ring array structure, and (**e**) topology design optimization procedure.

The topological design optimization scheme adopted in the present study was initially developed in our previous contribution considering the mutual coupling among acoustic, electric and mechanical systems. The magnitude of the sound pressure and the location of the focus point in the acoustic domain were defined as shown in Fig. 2a, and topology optimization for the piezoelectric rings was performed in the structure domain. The optimal design, shown in Fig. 2b, indicates that an MPRA with nine rings with different widths is required. For an operating frequency of 1 MHz and an applied voltage of 15 V, the sound pressure at the specified position is shown in Fig. 2c. The position of the maximum sound–pressure-focusing beam occurred at 10λ from the MPRA surface and coincided with the lateral length of the 3.3λ focusing beam, as shown in Fig. 2c. Due to the limitation of the manufacturability, the ring design going through in the thickness direction is hard to manufacture and has some limitations in experiments. Therefore, a plate was included with the piezoelectric substrate, in addition to the circular ring array. However, there was little difference between the acoustic pressure focusing responses of the MPRA with and without the plate, as shown in Fig. 2d. The topology optimization is presented in Fig. 2e. The material properties and the boundary conditions are assigned considering the experimental conditions. The thickness of the piezoelectric ring array plate is set to 0.5 mm. The actuation frequency is 1 MHz and the supply voltage is 15 V. The material properties of water are assigned. The final structure of the PUT tested in this study consists of a matching layer, an MPRA layer, a backing layer, and housing. With this structure, the matching layer is used to improve energy transfer from piezoelectric to acoustic media. Hence, to design an ultrasonic transducer that propagates waves in water, the material used for the matching layer must have similar properties to those of water. The backing plate on the rear of the MPRA was placed on this surface to support the PUT. As the focusing efficiency and signal-to-noise ratio depend on the acoustic impedance of this plate, an appropriate selection of backing materials is required. In addition, the attenuation coefficient of the backing material should be as high as possible to ensure that acoustic waves transmitted to the rear of the plate are not reflected.

**Figure 3:**
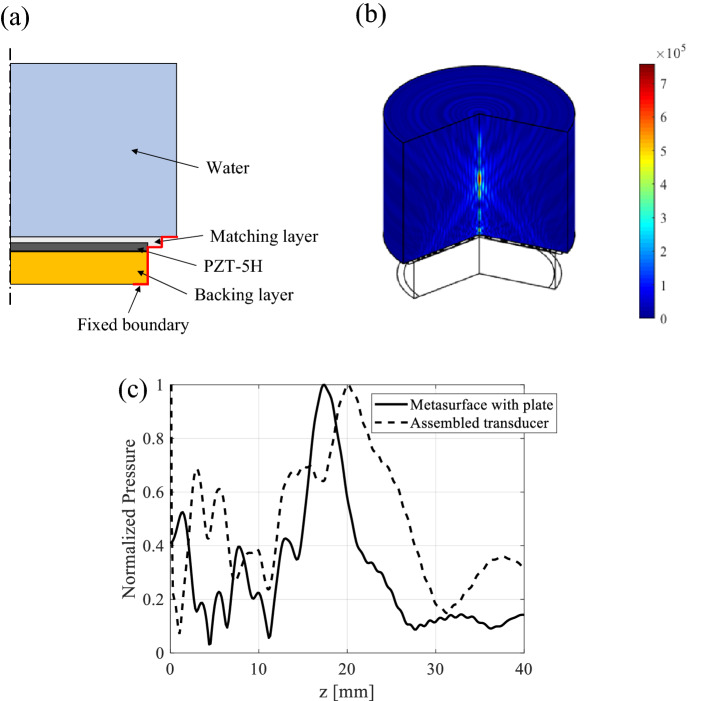
Finite element analysis of the PUT: (**a**) geometry of the 3D finite element model, (**b**) ultrasonic-focusing beam generated by the PUT in simulation, and (**c**) comparison of acoustic pressure focusing response of the PUT and the MPTA.

For a detailed analysis of the parameters of the PUT, we conducted a multiphysics simulation using COMSOL software, with the finite element model shown in Fig. 3a. Here, the property information of MPRA layer, matching layer and backing layer is used for multiphysics finite element analysis. The density, Poisson’s ratio and dielectric constant of MPRA layer (PZT-5H, IStec Co.) are 7600 kg/m^3^, 0.3 and 1900, respectively. Also, the piezoelectric properties, d^33^, d^31^ and d^15^ of MPRA layer are 411, − 202 and 498 pm/v, respectively. For the impedance matching between water and piezoelectric ring array, the density, Poisson’s ratio and Young’s modulus of the impedance matching materials (Stycast 2057, Henkel Co.) are 1580 kg/m^3^, 0.45 and 1.7 GPa, respectively. Also, in order to absorb the ultrasonic waves propagating to the rear surface of the piezoelectric ring array, the density, Poisson’s ratio and sound of speed of backing materials (Low-density epoxy, Epoxy tech., Inc.) are 618.5 kg/m^3^, 0.45 and 2020 m/s, respectively. As shown in Fig. 3b, the ultrasonic focusing beam of the assembled model was confirmed through simulation. As shown in Fig. 3c, the acoustic focusing simulation result of the assembled PUT model was compared with the MPRA model including only the piezoelectric ring array. The acoustic focusing position and width of the two models are different, which was analyzed as a problem in which acoustic waves generated from the MPRA are reflected by the impedance mismatch of the backing layer. However, the pressure of the acoustic focusing beam of the two models was similar behavior according to time change.

## Fabrication of device

**Figure 4:**
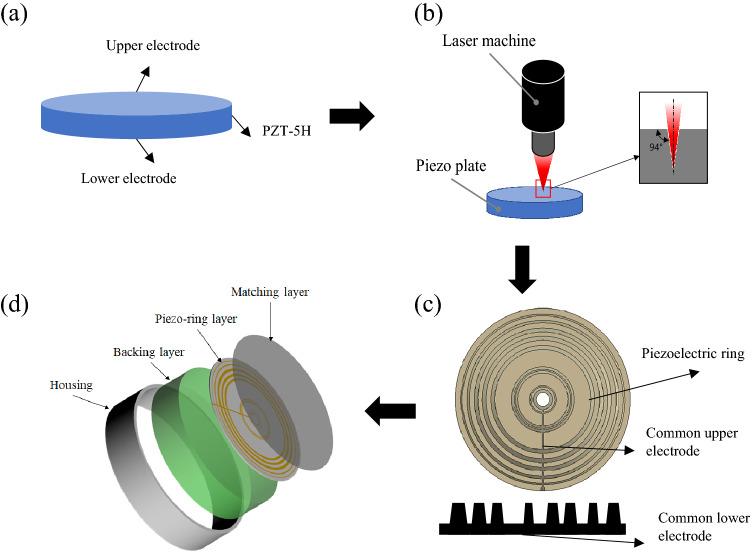
Fabrication and assembly of the MPRA-based PUT: (**a**) piezoelectric disk before processing. (**b**) laser ablation process. (**c**) patterned MPRA layer. (**d**) PUT packaging.

A summary of the fabrication and assembly process employed in this study for the creation of the PUT is included in Fig. 4. Here, we used laser ablation as a simple and efficient way to define the complex pattern for MPRA. This device consists of a circular 0.5-mm-thick piezoelectric substrate with a diameter of 45 mm. Electrodes are attached to the upper and lower surfaces of this substrate, as shown in Fig. 4a. A ring array pattern with a depth of 0.33 mm was inscribed on the piezoelectric substrate using a picosecond UV laser, as shown in Fig. 4b. The inclination of the cross-section of the ring structure with respect to the vertical axis was approximately 94° owing to the characteristics of the laser beam. A schematic of a processed MPRA for focusing on sound waves is shown in Fig. 4c. As the upper electrode is connected to each ring, it supplies a common driving voltage to the pattern, while the lower electrode is connected to a common ground. Arranging the MPRA in this manner makes the in-package supply of driving voltage to the upper and lower electrodes of the piezoelectric ring easy. An exploded diagram summarizing the assembly concept for the PUT is shown in Fig. 4d.

**Figure 5:**
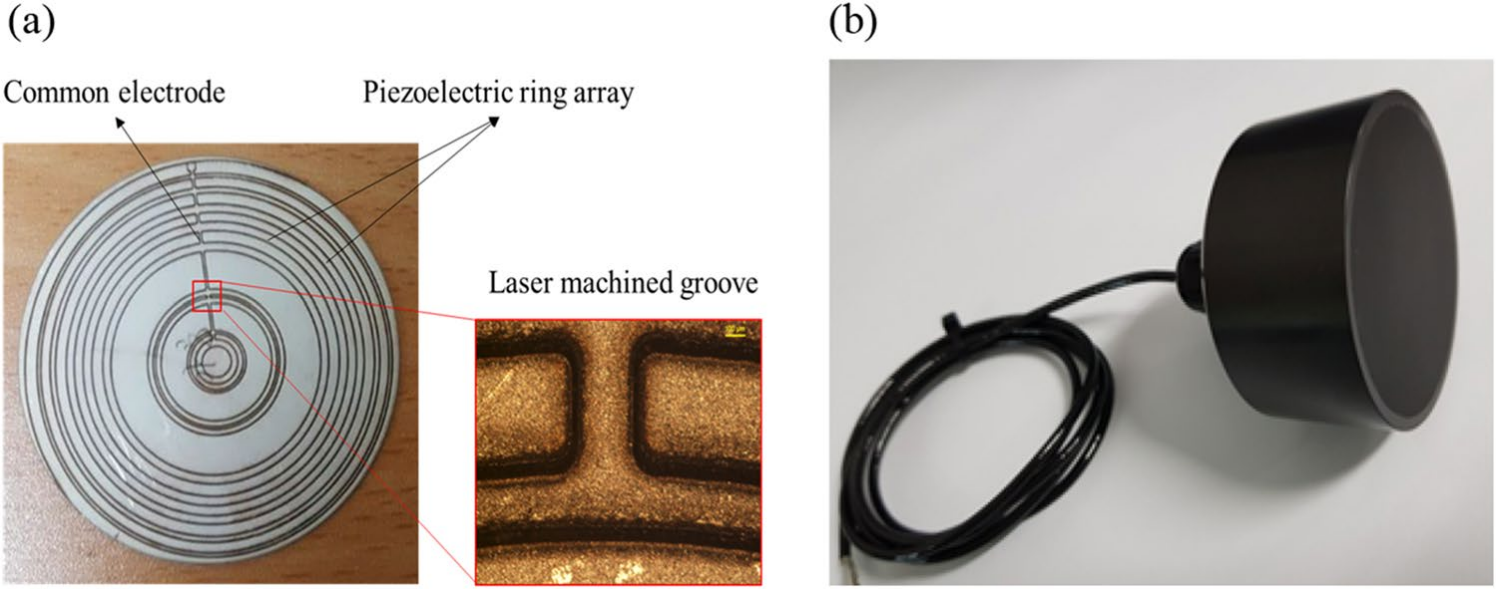
Photographs of (**a**) the MPRA and (**b**) a prototype of the MPRA-based PUT.

A photograph of the actual MPRA fabricated using laser ablation is shown in Fig. 5a, while the fully assembled ultrasonic-focusing transducer for subwavelength imaging is shown in Fig. 5b. In the latter image, the components were placed in a waterproof package for underwater operation.

## Measurement of acoustic pressure

Despite design efforts, a fabricated transducer’s focusing ability may not match the design specifications exactly owing to the acoustic, electrical, and structural properties of its constituent materials deviating from ideal behavior. Hence, we practically evaluated the electroacoustic properties of the focusing ultrasonic transducer by measuring the acoustic pressure at the point of focus and the spatial distribution of this pressure according to the voltage, frequency, and waveform of the input electrical signal. These electroacoustic properties are typically measured using a hydrophone with an active element (an element that outputs an electrical signal in response to sound waves) with a diameter similar to or smaller than that of the acoustic wavelength^35–37^.

**Figure 6:**
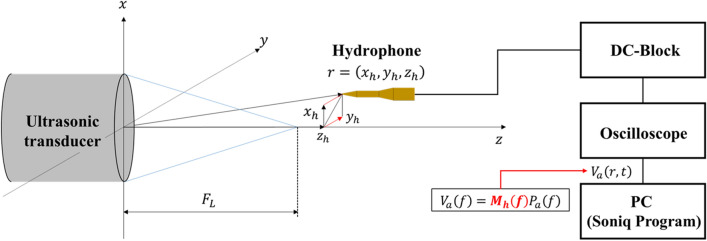
Configuration of representative devices for measuring the spatial and coordinates.

Figure 6 depicts a schematic diagram of an equipment configuration that receives ultrasound emitted from a transducer by a hydrophone. The hydrophone position was controlled by system control software (Soniq 5.1 Onda Co. Ltd.), which collects the time-domain signal from the oscilloscope and converts it into acoustic pressure. The acoustic pressure is coupled with the hydrophone position, $r=\left({x}_{h},{y}_{h},{z}_{h}\right)$, and finally transformed to a spatial distribution. The response of the voltage output to the input sound pressure reflecting the load impedance of all connected devices from the hydrophone to the oscilloscope is called the end-of-cable loaded sensitivity. The sensitivity of a hydrophone, ${M}_{h}$, is generally frequency dependent and is expressed by the following equation:

1 $${V}_{a}\left(f\right)={M}_{h}\left(f\right){P}_{a}\left(f\right),$$

where ${V}_{a}\left(f\right)$ denotes the spectral electrical signal, ${M}_{h}\left(f\right)$ denotes the end-of-cable-loaded sensitivity, and ${P}_{a}\left(f\right)$ denotes the acoustic pressure spectrum. For a tone-burst electrical signal, the acoustic pressure signal in the time domain can be approximately obtained as follows^37^:

2 $${P}_{a}\left(r,t\right)\approx {V}_{a}\left(r,t\right)/{M}_{h},$$

where ${P}_{a}\left(r,t\right)$ and ${V}_{a}\left(r,t\right)$ indicate an acoustic pressure signal and a voltage signal in the time domain, respectively. ${M}_{h}$ denotes the sensitivity at the specific frequency of interest.

## Device characterization

### Measurement setup for PUT characterization

**Figure 7:**
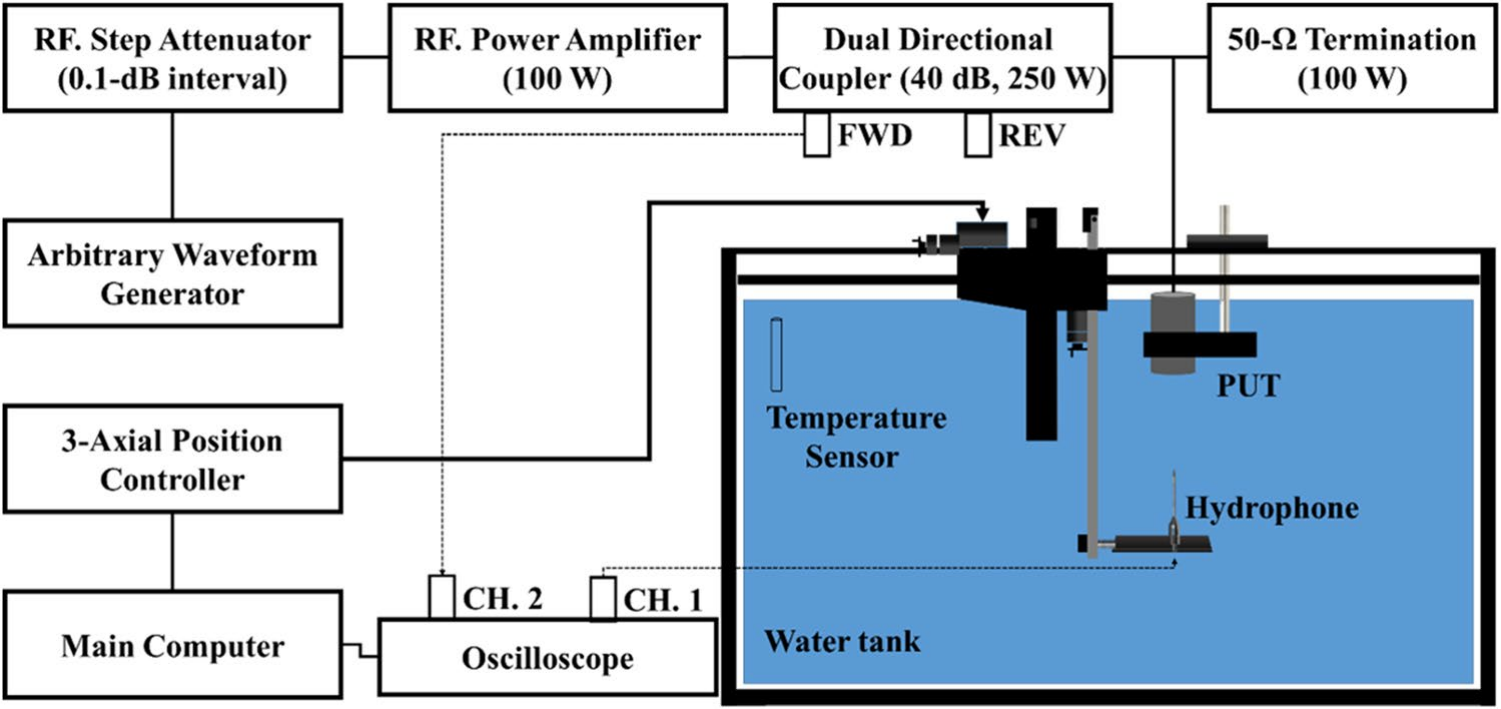
Configuration of the measurement setup used for characterizing the ultrasonic transducer.

Figure 7 depicts a system block diagram of the spatial distribution measurement via the miniature hydrophone scanning method. For electroacoustic characterization, the electrical input to the transducer and the output from the hydrophone are measured using a dual-channel digital oscilloscope (Model: DSO7012B, Agilent Co.). Here, Channel 1 of the oscilloscope is connected to the output terminal of the hydrophone (HNA0400, Onda Co.) through a DC block (AH-2010-DBCN, Onda Co.). Channel 2 is connected to the function generator through the J3 (FWD direction) terminus of the dual-directional coupler (C-5086-13, 0.01–250 MHz, 250 W, 40 dB, Werltone Co.). From the J3 terminal to Oscilloscope, the electric connection is made by using an approximately 2-m-long electric cable (RG58C/U, 2249-C-72 Pomona Co.) and 50 Ω feedthrough (Model 4119, 2W, Pomona Co.). An arbitrary waveform generator (33250A, Agilent Co.) is used to generate a 5-cycle tone-burst signal with a pulse repetition time of 1 ms. The generated signal is applied to an RF-Step Attenuator. The RF-Step Attenuator controls the magnitude of the input electric signal to the RF Power Amplifier with a 0.1-dB interval. The RF Power Amplifier (Model: 2100 L, ENI Co.) amplifies the signal sufficiently large to generate ultrasound into the water by PUT. A 50 Ω termination (100W, Model: RAU.200.0019.02, Rohde & Schwarz Co.) is connected in parallel with the ultrasonic transducer to prevent amplifier damage by reducing the practical electric impedance load. The acoustic pressure field can be obtained by the aforementioned method.

### Calibration of applied signal voltage to PUT

**Figure 8:**
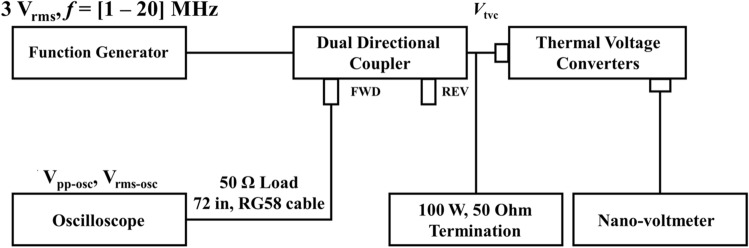
A setup of the frequency response calibration of the electric network by using a thermal voltage converter and oscilloscope for voltage correction applied to the ultrasonic transducer.

The dual directional coupler in Fig. 7 is used to monitor the voltage applied to the PUT with a 40 dB attenuation through the FWD terminal. Correction is necessary because the magnitude of the monitored electrical signal and that of the actual applied electrical signal are not the same. The correction is made through calibration of the electrical network placed in the middle. The calibration is a measurement of the ratio of the actual electrical signal voltage to the monitored electrical signal voltage as a function of frequency. Figure 8 shows the setup for calibrating the network. Here, the root mean square (RMS) voltage of the sinusoidal signal by the signal generator is set to be fixed at 3 V. The response $R$ can be obtained using the following equation.

3 $$R={V}_{tvc}/{V}_{rms-osc},$$

where ${V}_{tvc}$ and ${V}_{rms-osc}$ are the RMS voltage at the output terminal of the dual-directional coupler measured with the thermal voltage converter and the RMS voltage measured by connecting the 2-m-long cable from the FWD terminal of the dual-directional coupler to the oscilloscope, respectively. The AC-DC response of the thermal voltage converter can be calibrated at the national metrology institute.

**Figure 9:**
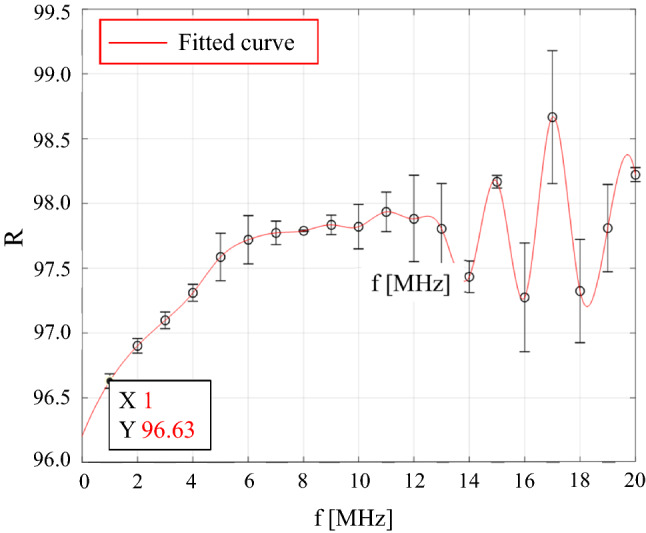
Correction value measurement result according to frequency.

In the system configuration of Fig. 7, the applied voltage to the front end of the ultrasonic transducer (${V}_{E}$) can be calculated by using the voltage measured by the oscilloscope (${V}_{osc}$) as follows.

4 $${V}_{E}=R\cdot {V}_{osc},$$

where $R$ is the correction factor at the specific frequency of interest (1.0 MHz), and has been measured to be 96.63. A graph plotting the variation of the correction factor with respect to frequency, according to Eq. (4), is included in Fig. 9.

## Results

**Figure 10:**
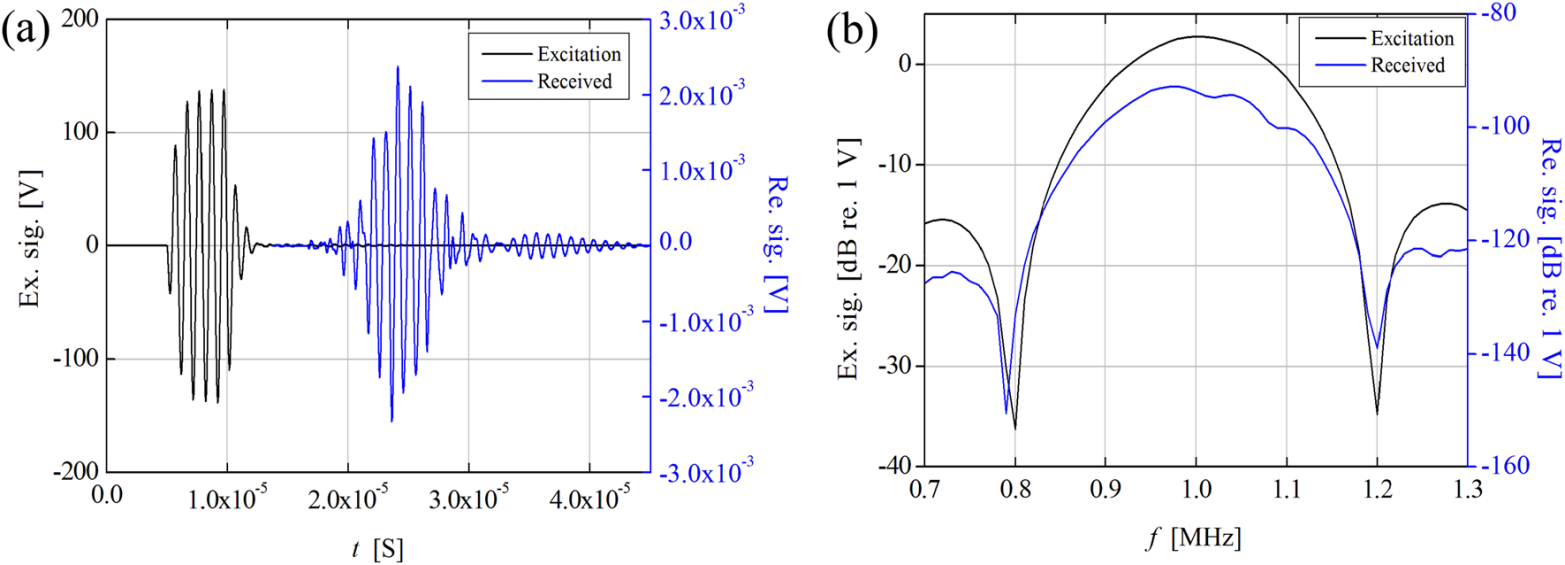
Measured acoustic response of the 1.0 MHz PUT: (**a**) pulse (black line) and echo waveforms (blue line), and (**b**) corresponding fast Fourier transform signals.

The PUT excitation voltage signal and the hydrophone received voltage signal are shown in Fig. 10. Figure 10a,b represent the time domain and frequency domain, respectively. As shown in Fig. 10b, the peak frequency of the spectrum of the excitation signal is 1.0 MHz, but that of the hydrophone received signal has a value slightly less than 1.0 MHz. This can be interpreted as being due to the frequency-dependent attenuation during the propagation of ultrasonic waves in water. In addition, when a 5-cycle tone-burst signal excitation is performed on the PUT, the − 6 dB bandwidth is approximately 37.5% based on 1.0 MHz. It is noteworthy that this is not exactly the same as the conventional pulser/receiver excitation bandwidth. Strictly speaking, the band width is the coupled characteristics of the pulse/receiver and the transducer.

**Figure 11:**
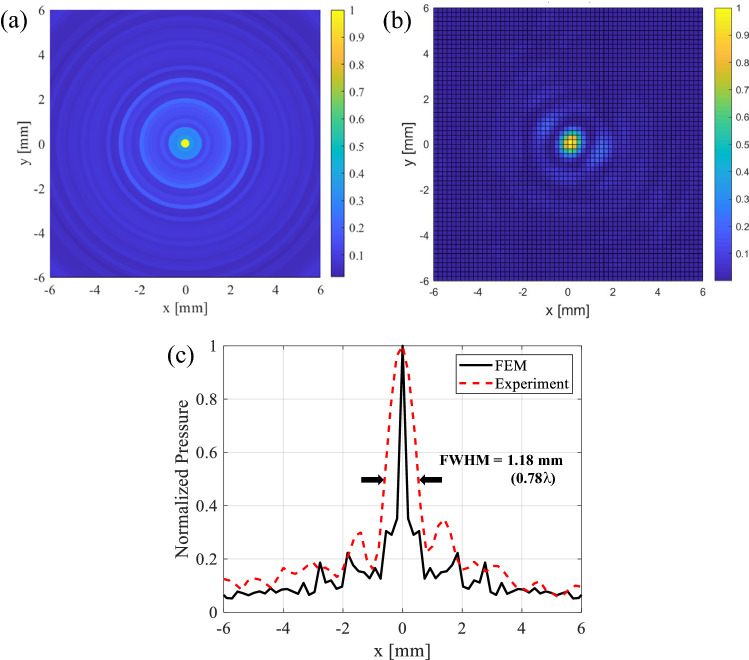
Characterization of the axial ultrasonic-focusing beam on the X–Y plane of the PUT: (**a**) normalized pressure field generated in simulation, (**b**) normalized pressure field measured in experiments, (**c**) comparison of the FWHM of the axial ultrasonic-focusing beams from simulation and experiment.

In the case of the x–y plane simulation result of the axially focused beam, the two-dimensional shape of the normalized acoustic pressure of the ultrasonic focused beam is shown in Fig. 11a. The full width at half-maximum (FWHM) of the axially focused beam was 0.19λ (0.285 mm), indicating good subwavelength ultrasonic focusing. In the case of the x–y plane experimental result of the axially focused beam, the full width at half-maximum (FWHM) of the axially focused beam was 0.78λ (1.18 mm) as shown in Fig. 11b. The difference in the FWHM between the simulation and experiment is confirmed by the shape of the ultrasonic focused beam along the x-axis direction, as shown in Fig. 11c.

**Figure 12:**
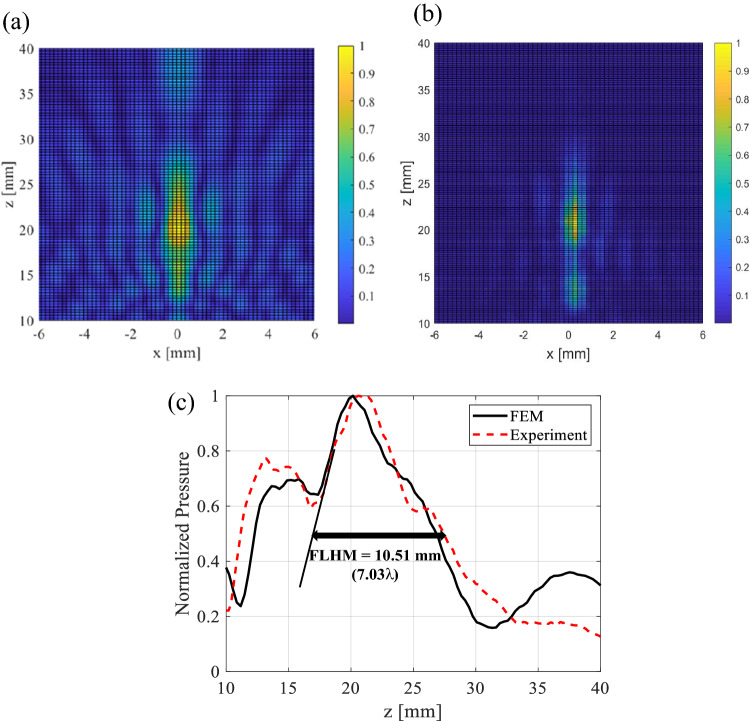
Characterization of the lateral ultrasonic-focusing beam on the X–Z plane of the PUT: (**a**) normalized pressure field generated in simulation, (**b**) normalized pressure field measured in experiments, (**c**) comparison of the FLHM of the lateral ultrasonic-focusing beams from simulation and experiment.

In the case of the x–z plane simulation and experimental result of the laterally focusing beam, the two-dimensional shape of the normalized acoustic pressure of the ultrasonic focusing beam is shown in Fig. 12a,b. For the acoustic pressure distribution of the lateral focus beam observed along the z-axis, the location at which the maximum pressure focus occurs is similar in simulations and experiments. As shown in Fig. 12c, the normalized acoustic pressure level of the lateral focal beam along the z-axis shows similar results between the simulation and experiment. From the simulation results, the undesirable focus point occurred at 7.46 λ (15.2 mm) from the z-axis origin. A main focus point occurred at 11.23λ (16.8 mm) from the z-axis origin, and the full lateral focus point at half-maximum (FLHM) of the lateral focus point was 4.04λ (6.048 mm). From the experimental results, the undesirable focus point occurred at a similar position as the simulation. The main focus point occurred at 10.89 λ (16.3 mm) from the z-axis origin, and the full lateral at half-maximum (FLHM) was 7.03 λ (10.51 mm).

## Discussion

In this study, we employed topology optimization based on a solid isotropic material with a penalization method to determine the optimal design for an MPRA that can form a needle-like subwavelength ultrasonic-focusing beam. Although this optimization method can define a structural design for realizing the target performance, this does not include detailed consideration of component elements. Accordingly, the final structure and performance of the MPRA-based PUT were refined through multiphysics finite element analysis. Following trials investigating optimal process conditions, an MPRA with a complex pattern was fabricated using a laser ablation process. Subsequently, a PUT capable of operating in water was fabricated by assembling the MPRA, matching layer, and backing layer in waterproof packaging. The driving frequency of the fully assembled PUT was determined to be 1.0 MHz in a characteristic test, and its − 6 dB frequency bandwidth was measured to be 37.5%. The subwavelength ultrasonic focusing performance was also characterized using a pulse-echo test. From this experiment, the FWHM of the axial ultrasonic-focusing beam was determined to be 0.78 λ, forming a needle-type subwavelength focusing beam, and the FLHM of the lateral ultrasonic-focusing beam formed at 10.89 λ from the transducer was 7.03 λ. Hence, the transducer developed in this study can form a needle-type subwavelength focusing beam, the first such achievement for a piezoelectric ring-array-based PUT. Based on this performance, this subwavelength ultrasonic-focusing technology displays potential for high-resolution imaging and medical ultrasonic technologies.

## Methods

### Laser ablation process

Lead zirconate-titanate (PZT-5H) disks with an optical density of 5, a diameter of 45 mm, and a thickness of 0.5 mm were used as the piezoelectric materials in this study. These disks were supplied with silver electrodes attached to their surface. The MPRA pattern was inscribed on the upper surfaces of these samples using a UV laser system (Pico 355, Kortherm Science Co., South Korea), with the laser pulse energy set at 3.0 µJ, the repetition rate at 15 kHz, and the scribing speed at 200 mm/s. The laser wavelength with this system is 355 nm, and each laser beam has a pulse width of 10 ps. Each inscription was completed with a total of 150 passes. A processing width of 30 µm was defined between each ring in the pattern, and the piezoelectric disk was inscribed to a depth of 330 µm. As the electrodes on the upper surface of the piezoelectric material were not removed by laser processing, the nine rings in the MPRA were all connected to a common upper electrode. Similarly, these rings are all connected to a common lower electrode, as the lower surface of the piezoelectric material was not processed.
